# Bacterial Superantigen Toxins, CD28, and Drug Development

**DOI:** 10.3390/toxins10110459

**Published:** 2018-11-06

**Authors:** Raymond Kaempfer

**Affiliations:** Department of Biochemistry and Molecular Biology, Institute of Medical Research Israel-Canada, The Hebrew University-Hadassah Medical School, Jerusalem 9112102, Israel; kaempfer@hebrew.edu; Tel.: +972-2-675-8389

**Keywords:** bacterial superantigen toxins, lethal toxic shock, costimulation, CD28 receptor, CD28 homodimer interface, B7-2 receptor, receptor dimer interface mimetic peptides

## Abstract

During severe bacterial infections, death and disease are often caused by an overly strong immune response of the human host. Acute toxic shock is induced by superantigen toxins, a diverse set of proteins secreted by Gram-positive staphylococcal and streptococcal bacterial strains that overstimulate the inflammatory response by orders of magnitude. The need to protect from superantigen toxins led to our discovery that in addition to the well-known MHC class II and T cell receptors, the principal costimulatory receptor, CD28, and its constitutively expressed coligand, B7-2 (CD86), previously thought to have only costimulatory function, are actually critical superantigen receptors. Binding of the superantigen into the homodimer interfaces of these costimulatory receptors greatly enhances B7-2/CD28 engagement, leading to excessive pro-inflammatory signaling. This finding led to the design of short receptor dimer interface mimetic peptides that block the binding of superantigen and thus protect from death. It then turned out that such a peptide will protect also from Gram-negative bacterial infection and from polymicrobial sepsis. One such CD28 mimetic peptide is advancing in a Phase 3 clinical trial to protect from lethal wound infections by flesh-eating bacteria. These host-oriented therapeutics target the human immune system itself, rendering pathogens less likely to become resistant.

## 1. Introduction

The ubiquitous Gram-positive bacterial strains, *Staphylococcus aureus* and *Streptococcus pyogenes*, secrete pyrogenic exotoxins called superantigens because they evoke a vastly exaggerated immune response that renders them lethal to humans [1]. These proteins hyperinduce inflammatory cytokines even at femtomolar concentrations (10^−15^ M). The superantigen family contains several dozen members that are structurally diverse; thus, staphylococcal enterotoxin B (SEB), a prominent staphylococcal superantigen, is only 6% homologous to staphylococcal toxic shock syndrome toxin-1 (TSST-1), the most remote member of the superantigen family [1]. Known to be not only a medical problem but also a potential biological weapon, the superantigens are classified as a Category B priority pathogen [2,3]. Bacterial strains can produce multiple superantigens having distinct structures [1], impeding the development of a broadly effective vaccine while emphasizing the need for broad-spectrum protection. 

Superantigen toxins use an unconventional mechanism to activate a strong T cell-mediated immune response. Whereas ordinary antigens are processed by the antigen-presenting cell and presented via its MHC class II (MHC-II) molecule to the T cell receptor (TCR) on the T cell, superantigens bind directly, as intact proteins, to the MHC-II molecule and the TCR on the cell surface, without need for antigen processing, bypassing thereby the restriction that limits T-cell activation by conventional antigens to some 0.01% of all T cells, as opposed to some 20% by the superantigens [4,5,6]. This classical view remained accepted for over two decades. It was the unexpected discovery that CD28 [7] and its B7-2 coligand [8], hitherto unknown to bind pathogens or virulence factors and thought to function only as the immune system’s principal costimulatory receptors [9,10], actually function as critical receptors for the superantigens. This in turn led to the characterization of these new superantigen-receptor interactions and to the design of short mimetic peptides that protect from lethal superantigen challenge [1,7,8] and indeed, from polymicrobial sepsis [11]. One such CD28 mimetic peptide shows promise as a drug against severe necrotizing soft tissue infections (‘flesh-eating bacteria’) [12] and is now advancing in a Phase 3 clinical trial.

## 2. CD28 and B7-2 Are Key Superantigen Receptors and Dimer Interface Mimetic Peptides Protect from Death

Effective activation of T cells, resulting in the induction of inflammatory cytokines, requires engagement of costimulatory receptor CD28 on the T cell with its B7-1 (CD80) and B7-2 (CD86) coligands on antigen-presenting cells. Expressed constitutively, CD28 is a homodimer that plays an essential role in activating both innate and adaptive immune responses [9,10,13,14]. B7-1 is induced only in the course of an immune response upon CD28 signaling, but B7-2 is expressed constitutively [14,15]; hence, the immediate inflammatory response is controlled by the B7-2–CD28 interaction [16,17].

In search of an antidote for superantigen toxicity, we noticed that a 12-amino acid β-strand/hinge/α-helix domain is conserved structurally within the family of diverse superantigens [1]. Notably, this domain, which had no known function, is far removed from the binding sites for MHC-II molecule and TCR and indeed is found on the opposite side of the superantigen protein molecule. Remarkably, peptide mimetics of this domain block superantigen toxicity in mice and attenuate the inflammatory cytokine response to superantigens in human peripheral blood mononuclear cells (PBMC) [1].

**Figure 1:**
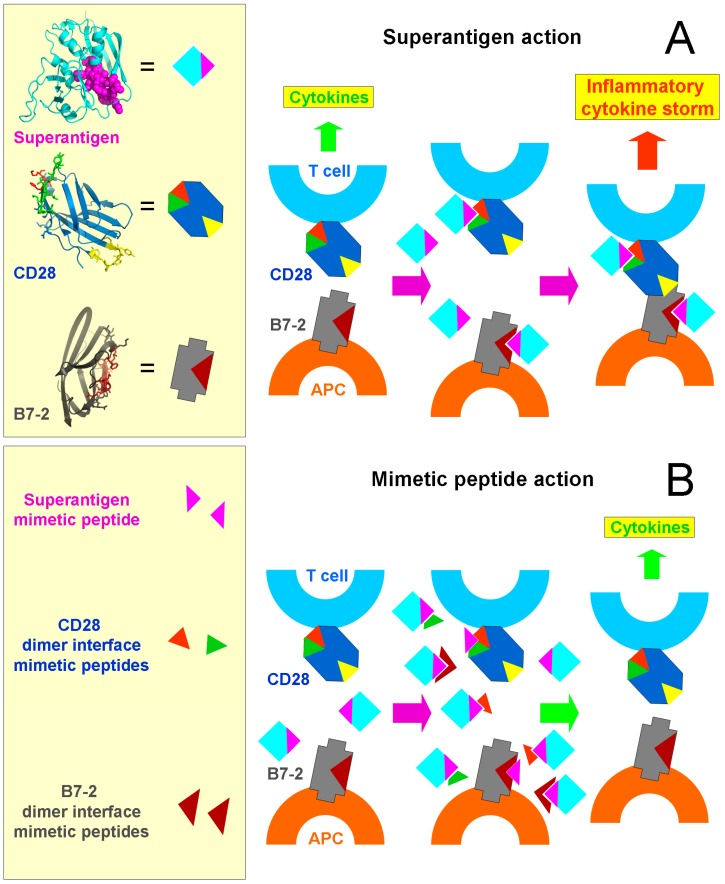
Mechanism of action of superantigen toxins and of mimetic peptide antagonists. (**A**) The superantigen acts to induce an inflammatory cytokine storm. In the prototypical superantigen SEB, the conserved β-strand/hinge/α-helix superantigen domain (magenta) points away from the binding sites for MHC-II molecule and TCR that were omitted for clarity; the extracellular domain of CD28 shows the homodimer interface (red and green) and the site where its B7 coligands bind (yellow) and the extracellular domain of B7-2 shows the crystallographic homodimer interface (brown). Normal immune stimulation leads to a moderate CD28–B7-2 interaction between antigen-presenting cell and T cell and to moderate cytokine induction (**left**). However, when the superantigen engages the homodimer interface of CD28 and of B7-2 (**middle**), this potently enhances CD28–B7-2 engagement, resulting in an inflammatory cytokine storm (**right**) [8]. (**B**) Short peptide mimetics of the β-strand/hinge/α-helix superantigen domain [1] or of the homodimer interfaces in CD28 [7] or in B7-2 [8] act as competitors that prevent access of the superantigen to its CD28 and B7-2 host targets (**middle**) and thereby abrogate excessive signaling through the CD28/B7-2 axis (**right**).

We speculated that this conserved yet remote domain might bind a novel and essential receptor. A monoclonal antibody against CD28 that induces inflammatory cytokines in human PBMC was inhibited by a β-strand/hinge/α-helix superantigen domain mimetic peptide [7]. Could the costimulatory receptor CD28 be a superantigen receptor? Indeed, even in the absence of MHC-II or TCR, superantigens bind directly to CD28 on the cell surface as shown by confocal microscopy of CD28-expressing cells and CD28 binds directly to the β-strand/hinge/α-helix domain mimetic peptide (Figure 1). Epitope mapping of the anti-CD28 monoclonal antibody led to the CD28 homodimer interface. Short (8–10 amino acid) peptides derived from the complex CD28 dimer interface, to which regions located over 100 amino acids apart contribute, bind superantigens directly, inhibit inflammatory cytokine induction by superantigens in human PBMC, and protect mice from lethal superantigen challenge [7] (Figure 1).

Why would CD28 be needed as superantigen receptor? Could there also be a role for its coligand B7-2? A similar experimental approach showed that this is indeed the case. More than that, even though B7-2 is a weak, noncovalent dimer that is mostly monomeric on the surface of the antigen-presenting cell [17,18], peptides derived from the crystallographic B7-2 homodimer interface, yet not peptides that fall outside its dimer interface, act as potent superantigen antagonists both in human PBMC and in superantigen-challenged mice and bind directly to diverse superantigens [8] (Figure 1B).

Of note, the superantigen uses the same conserved β-strand/hinge/α-helix domain [1] to bind to both CD28 and B7-2. SEB mutated at two positions within this domain fails to bind either CD28 [7] or B7-2 [8] and lacks toxicity and the ability to induce inflammatory cytokines. The superantigen synapse between antigen-presenting cell and T cell thus involves two superantigen molecules that each engage the MHC-II molecule and TCR on one side of the protein, and on their other side the dimer interface of either CD28 or B7-2 [8]. The question now became: why is there a need for this dual binding?

## 3. The Superantigen Triggers B7-2/CD28 Costimulatory Receptor Engagement

The interaction between CD28 and its B7-2 coligand is weak, in the 20 micromolar range of affinity [19]. This ensures a moderate signal for the inflammatory response, sufficient to promote protective immunity yet avoiding a harmful inflammatory cytokine storm (Figure 1A). To examine the question, whether binding of the superantigen to CD28 and B7-2 might influence their interaction, we used three independent assays: (*i*) binding of soluble B7-2 to CD28 expressed on the surface of transfected cells in the absence of any MHC-II molecule or TCR, quantitated by western blotting; (*ii*) conversely, binding of soluble CD28 to B7-2 expressed on the surface of transfected cells in the absence of any MHC-II molecule or TCR; and (*iii*) synapse formation between CD28 and B7-2, both expressed on the surface of transfected cells in the absence of any MHC-II molecule or TCR, through use of flow cytometry [8]. In this manner, the B7-2–CD28 interaction could be quantitated in the absence of confounding multiple ligand-receptor interactions that contribute to synapse formation between antigen-presenting cell and T cell, involving not only MHC-II/TCR but additional costimulatory ligand pairs whose expression could be affected by superantigen exposure, resulting in changes in synapse strength.

This approach validated that the superantigen strongly enhances B7-2–CD28 engagement [8]. Thus, it is the formation of this costimulatory axis between antigen-presenting cell and T cell that critically controls the strength of the inflammatory response. By enhancing this key interaction, superantigens induce a cytokine storm (Figure 1A). That is a surprising result in view of the fact that the point of engagement of superantigen within CD28 and within B7-2 involves in both cases not the site where the co-receptors interact but instead, the homodimer interface which is remote and indeed located at the opposite pole within the extracellular domain of CD28 [20,21]. 

## 4. Bacterial Infection

The work on superantigens raised the question, whether peptides that protect from lethal superantigen challenge might also protect from severe Gram-positive live bacterial infection. Indeed, mice were protected from live, superantigen-producing streptococcal infection [22]. Moreover, a CD28 dimer interface mimetic peptide inhibits the induction of tumor necrosis factor or interleukin-6 in human PBMC by lipopolysaccharide (LPS), a hallmark of Gram-negative bacteria, and protects mice from lethal challenge with LPS as well as from *E. coli* peritonitis and from polymicrobial sepsis elicited by cecal ligation and puncture [11]. This indicates a broad-spectrum mode of action of the peptide. Indeed, as already mentioned, such a peptide is now advancing in a Phase 3 clinical trial in necrotizing soft tissue infection, where death can often be avoided only by amputation of a limb or excision of a large amount of flesh, strongly traumatic clinical end points in a disease currently without a drug [12]. How the peptide prevents inflammatory cytokine storm in such cases (which often involve streptococci) is presently under investigation.

## 5. Conclusions

Driven initially by the need to protect from superantigen toxins as potential biological weapons that cause lethal toxic shock, this research has led to the identification of formation of the CD28–B7-2 costimulatory axis as a major checkpoint for toxicity. Superantigens bind into the homodimer interface of CD28 and of B7-2, regions remote from where these two receptors interact, yet it is the binding of superantigen that triggers the CD28–B7-2 interaction (Figure 1A). To induce an inflammatory cytokine storm, superantigens thus subvert the costimulatory axis. Mimetic peptides derived from the CD28 or B7-2 homodimer interface are host-oriented therapeutics that target the human immune system, rather than the pathogen (Figure 1B). For that reason, pathogens cannot readily become drug-resistant, given that the immune system and its receptors will not change in our lifetime and well beyond.
